# To Our Subscribers

**Published:** 1877-04-20

**Authors:** 


					﻿Editorial and Miscellaneous.
TO OUR SUBSCRIBERS.
It is believed that not more than one
physician in ten takes and pays for a
medical journal. The result is that
scarce one in ten keep pace with the pro-
gress of the profession, or contribute
anything to its advancement. This is
unfortunate and wrong. We, as journ-
alists, are doing all that we can to induce
the members of the profession to read,
to improve themselves, and to push for-
ward the car of medical progress. But
we labor under the difficulty of not be-
ing able to see and talk with them per-
sonally. Appeals made through our
journal they do not see, and circulars
sent out are too often not read, or at-
tract but transcient notice. We believe
that there are thousands of practitione/s,
who, if talked to, personally, in regard
to the importance of medical journals
and their duty to read and keep up with
the advancement of the profession,
could be readily induced to subscribe
for the Record. We, therefore, again
urge our subscribers, as we. have often
done before, to think of this matter,
and aid us in extending our circulation.
Every man has some friend or acquaint-
ance whom he can influence to sub-
scribe. We earnestly request our friends
to help us. We have certain improve-
ments in contemplation for our journal
which will accrue to the benefit of our
readers, but we need a large list of sub-
scribers to be able to accomplish them.
Take hold then, friends, and work for
us. It will help us, and enable us the
better to serve you. It will benefit
those whom you induce to subscribe.
It will tend to elevate the profession,
and wiU promote the public good.
				

